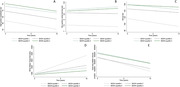# Social determinants in early midlife and trajectories in cognition and brain integrity in midlife

**DOI:** 10.1002/alz.091709

**Published:** 2025-01-09

**Authors:** Christina S. Dintica, Julia Cheunkarndee, R. Nick Bryan, Lenore J. Launer, Kristine Yaffe

**Affiliations:** ^1^ University of California, San Francisco, San Francisco, CA USA; ^2^ Northern California Institute for Research and Education, San Francisco, CA USA; ^3^ University of Pennsylvania, Philadelphia, PA USA; ^4^ National Institute on Aging, Baltimore, MD USA; ^5^ University of California San Francisco / San Francisco VA Medical Center, San Francisco, CA USA

## Abstract

**Background:**

Social determinants of health (SDOH) are increasingly recognized as important drivers of inequities in cognitive outcomes. However, most existing evidence is based on individual SDOH components. We evaluated the relationship between a cumulative SDOH index in early midlife and change in cognition and brain volume.

**Methods:**

We included 3,224 participants from the Coronary Artery Risk Development in Young Adults (CARDIA) study with information on SDOH in early midlife (mean age 40.0 ± SD 3.6) and cognitive assessments and brain MRI 10, 15, and 20 years later. A weighted SDOH score representing the cumulative number of unfavorable SDOHs, identified from 13 components across 4 domains (economic stability, community and social context, education, and health care system access) was calculated and divided into quartiles (quartile 1 being the least favorable). Cognition was assessed using the Digit Symbol Substitution Test (DSST), the Stroop Test, and the Rey Auditory Verbal Learning Test (RAVLT). 1,108 participants had MRIs measuring white matter hyperintesities (WMHs) and total gray matter (GM). Mixed linear regression was used to examine the association between the SDOH index quartiles and cognitive and MRI outcomes adjusting for age, sex, race, and intracranial volume (for MRI).

**Results:**

Participants in the least favorable SDOH quartile had steeper decline in the Stroop test (b: ‐0.16, 95% CI: ‐0.31 to ‐0.02) and the RAVLT (b: ‐0.04, ‐0.08 to ‐0.01), but not the DSST (b: ‐0.08, ‐0.21 to 0.07), compared to the most favorable SDOH quartile (Figure 1, A‐C). Moreover, participants in the least favorable SDOH quartile had higher accumulation of WMHs over time (b: 196.31, 95% CI: 89.41 to 303.21) and a steeper decline in total GM (b: ‐565.10, 95% CI: ‐1021.52 to ‐108.69), compared to quartile 4 (Figure 1, D, E). Quartiles 2 and 3 were not significantly different in rates of change in any of the cognitive tests or MRI measures compared to quartile 4.

**Conclusion:**

High social disadvantage in early midlife is associated with accelerated decline in cognition and worsening of brain integrity already at midlife. Further studies identifying underlying mechanisms linking SDOH with cognition are needed to establish prevention strategies.